# Cardiac Tamponade Secondary to Hemorrhagic Pericardial Effusion: A Complication of STEMI

**DOI:** 10.31486/toj.23.0023

**Published:** 2023

**Authors:** Marvin Kajy, Amy Rechenberg, Connor Kerndt, Kevin Wolschleger

**Affiliations:** ^1^ Department of Cardiology, Spectrum Health, Michigan State University, Grand Rapids, MI; ^2^ Department of Internal Medicine, Spectrum Health, Michigan State University, Grand Rapids, MI; ^3^ Department of Cardiology, Spectrum Health, Grand Rapids, MI

**Keywords:** *Cardiac tamponade*, *COVID-19*, *heart rupture*, *myocardial infarction*, *pericardial effusion*

## Abstract

**Background:** Most pericardial effusions that occur in the setting of ST-segment elevation myocardial infarction (STEMI) are small, simple, and without symptomology. However, in its most severe form, pericardial effusion can precipitate cardiac tamponade, and when untreated, can cause abrupt hemodynamic instability. Pericardial effusion may be a manifestation of left ventricular free-wall rupture, hemorrhagic pericarditis, or aortic dissection involving a coronary artery.

**Case Report:** We describe the case of a 65-year-old male who experienced chest pain for several days prior to admission but delayed seeking care because he wished to avoid coronavirus disease 2019 exposure. Upon arrival, he was hemodynamically unstable. Electrocardiogram was consistent with anterior STEMI. Bedside echocardiogram demonstrated a hypertrophic left ventricle with preserved function and a large, complex pericardial effusion with cardiac tamponade physiology. Computed tomography of the chest identified hemopericardium but was unable to delineate etiology. The patient underwent emergent thoracotomy because of persistent shock, and during the surgery, left ventricular free-wall rupture was identified and repaired. Coronary artery bypass grafting to the patient's left anterior descending artery was also performed. The patient remained asymptomatic at 2-year follow-up.

**Conclusion:** The differential for hemodynamic compromise in a patient with STEMI is broad, but quickly distinguishing pump failure from other life-threatening causes of shock is imperative to dictate time-sensitive management decisions. The presence of a hemorrhagic pericardial effusion in the setting of STEMI is a surrogate marker for a severe infarct and can help the bedside physician determine whether a patient will be better served in the catheterization lab for revascularization or in the operating room for surgical repair.

## INTRODUCTION

Pericardial effusion is a common echocardiographic finding. The Framingham Heart Study suggests that pericardial effusion may be present in approximately 6.5% of the general adult population,^1^ but in higher risk populations, the incidence may be as high as 13% to 20%, as described in high-risk emergency departments.^1,2^ The incidence of pericardial effusion has been demonstrated to increase with age,^1^ approximating 20% in patients >80 years of age vs <1% in individuals 20 to 30 years of age.^1^

Pericardial effusion is classified as simple or complex, depending on the consistency of the pericardial fluid. Most pericardial effusions are simple and small, do not cause hemodynamic compromise, and frequently result in no significant physiologic alterations or symptomology. They occur as a result of pericardial inflammation and increased microvascular permeability inside the necrotic myocardial tissue.^3^ In its most severe form, a pericardial effusion can precipitate cardiac tamponade, and when untreated, can cause abrupt hemodynamic instability. Pericardial effusion is a common complication of ST-segment elevation myocardial infarction (STEMI)^3^ and may be a manifestation of left ventricular free-wall rupture (LVFWR), hemorrhagic pericarditis, or aortic dissection involving a coronary artery.^4^ LVFWR has become a rare complication of STEMI in the percutaneous coronary intervention era because of prompt and effective revascularization.^5^ However, the incidence of LVFWR and other mechanical complications of STEMI increased during the coronavirus disease 2019 pandemic as patients delayed seeking care out of fear of contracting the virus in the medical setting.^6,7^

Providers should be aware of a pericardial effusion in the setting of STEMI, as a hemodynamically significant pericardial effusion has a significant impact on treatment approach. Patients with STEMI and benign pericardial effusion may safely proceed to the catheterization laboratory for revascularization. However, for patients with STEMI and pericardial effusion causing tamponade, clinicians must attempt to characterize the pericardial effusion and delineate its etiology. In the setting of STEMI and hemorrhagic pericardial effusion, surgical exploration may be necessary to elucidate the mechanism of pericardial effusion and appropriately treat the underlying disease.

We describe the case of a patient with STEMI and concomitant pericardial effusion secondary to early LVFWR that was identified rapidly and successfully repaired surgically.

## CASE REPORT

A 65-year-old male with a medical history of hypertension, tobacco use disorder, and obesity presented with substernal, nonpositional, nonradiating chest pain that had started 4 to 5 days prior. The patient had no other known cardiovascular risk factors. His chest pain, which he also self-described as chest tightness, acutely worsened 35 minutes prior to arrival with newly associated dyspnea, dizziness, and weakness. His weakness and pain provoked a collapse to the floor without loss of consciousness and prompted him to seek emergency medical services.

On presentation, the patient was hypotensive (blood pressure 77/60 mm Hg), tachycardic (heart rate 110 beats/min), and tachypneic (respiratory rate of 21 breaths/min), and he had a temperature of 36.7 °C (98.1 °F). Physical examination revealed the patient to be in moderate distress. Lungs were clear to auscultation. Heart sounds were difficult to appreciate. The patient had jugular venous distention up to 12 cm, and his radial, dorsalis pedis, and posterior tibial pulses were diminished bilaterally. On integumentary examination, the patient demonstrated cold extremities with a mottled appearance of the head, chest, and bilateral upper extremities concerning for shock.

**Figure 1.**
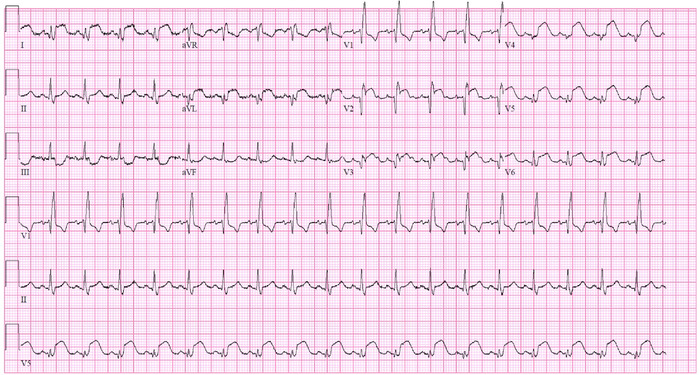
Electrocardiogram at presentation demonstrated ST-segment elevation in leads V2 through V6 with reciprocal ST-segment abnormalities in the inferior leads and right bundle branch block.

**Figure 2.**
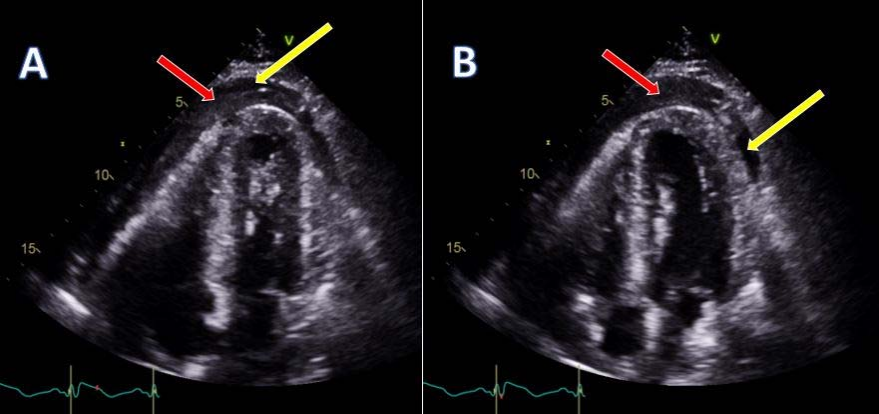
Transthoracic echocardiogram in apical 4-chamber view in (A) systole and (B) diastole. Yellow arrows point to the pericardial effusion, and red arrows point to echo-dense coagulum within the pericardial effusion.

**Figure 3.**
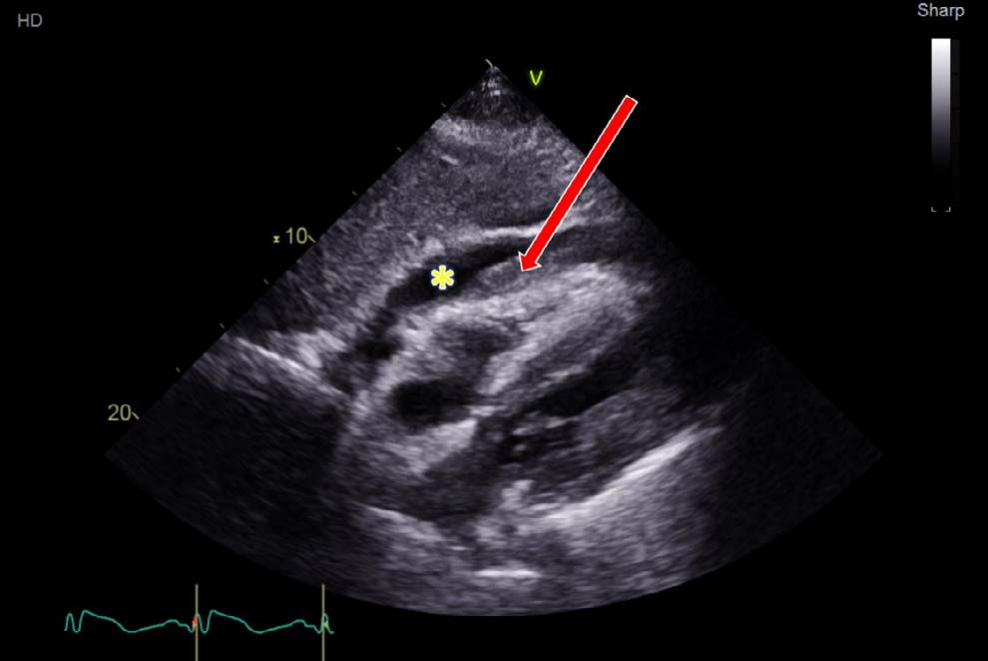
Transthoracic echocardiogram in subcostal view. The yellow asterisk denotes the pericardial effusion, and the red arrow points to echo-dense coagulum within the pericardial effusion.

Electrocardiogram revealed ST-segment elevation in leads V2 through V6 with reciprocal ST-segment abnormalities in the inferior leads and right bundle branch block (Figure 1). High-sensitivity troponin was elevated to 1,563 ng/L (reference range, <22 ng/L). Bedside echocardiogram demonstrated preserved left ventricular function with a moderate size pericardial effusion with echo-dense characteristics (Figures 2 and 3). Given the patient's undifferentiated shock, he was administered 3 L of crystalloid fluids and 1 unit of uncrossed red blood cells while he was in the trauma bay. The patient was persistently hypotensive and was started on vasopressor support, initially with norepinephrine and then with epinephrine to stabilize his shock before computed tomography (CT) evaluation. Urgent chest CT identified hemopericardium but showed no clear etiology. The differential at that time was concerning for aortic dissection extending retrograde into the left main/left anterior descending artery or LVFWR associated with old infarction in the left anterior descending artery.

**Figure 4.**
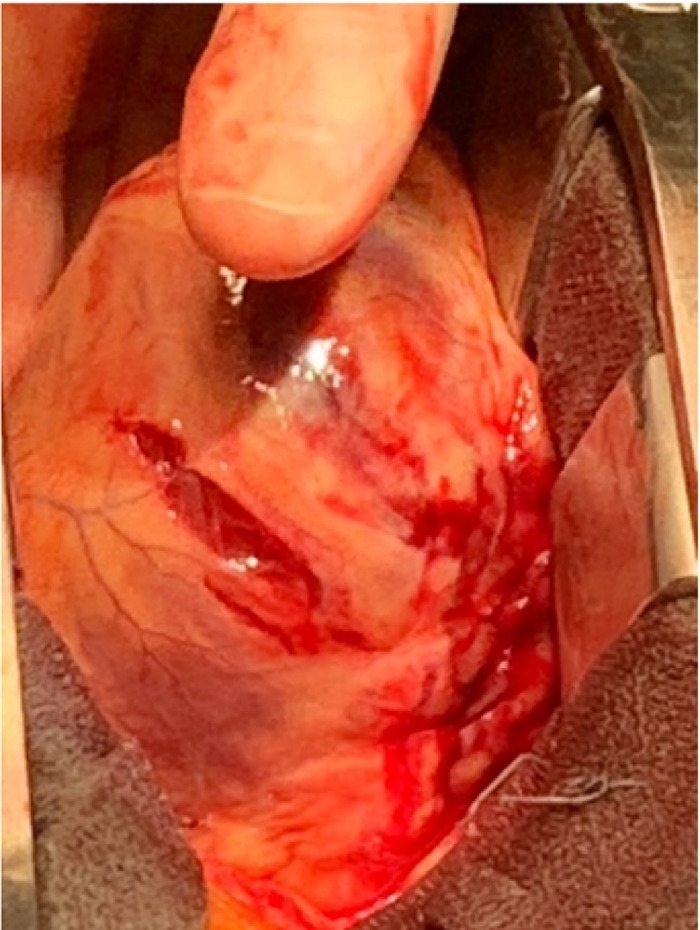
The patient had a deep laceration and an adjacent smaller laceration along the anterolateral wall of the left ventricle.

**Figure 5.**
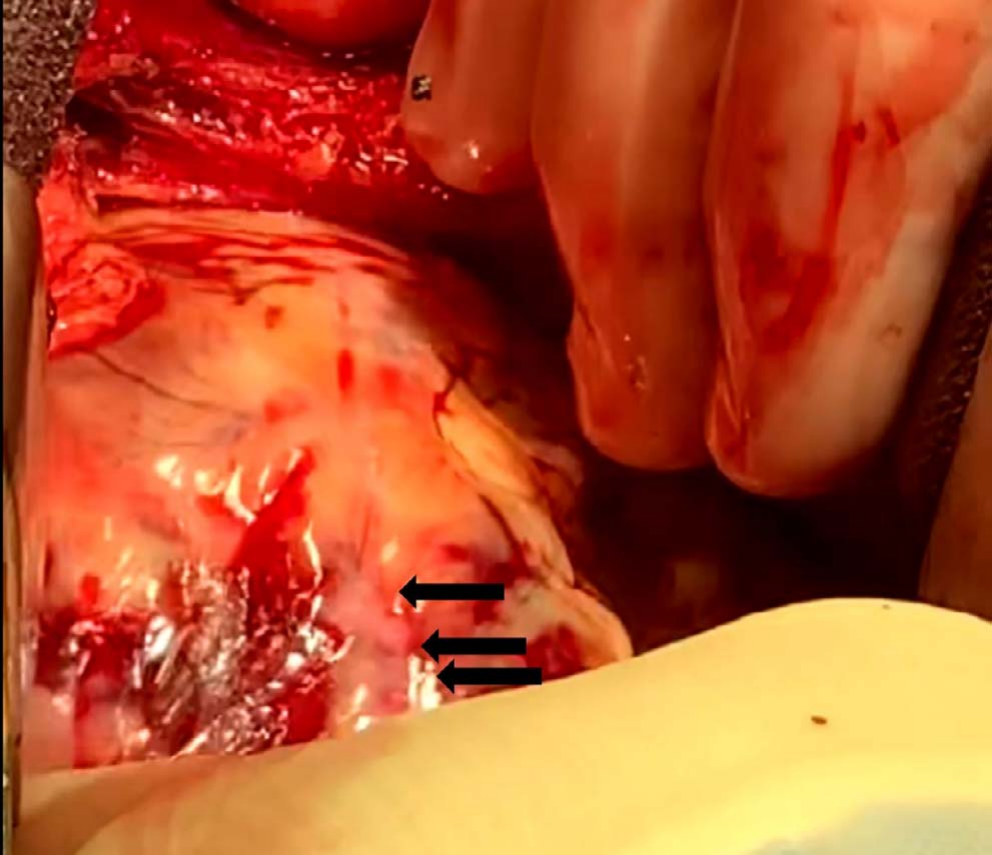
Edematous petechiae stained pattern of the left anterior descending artery (black arrows) was consistent with an acute infarct.

**Figure 6.**
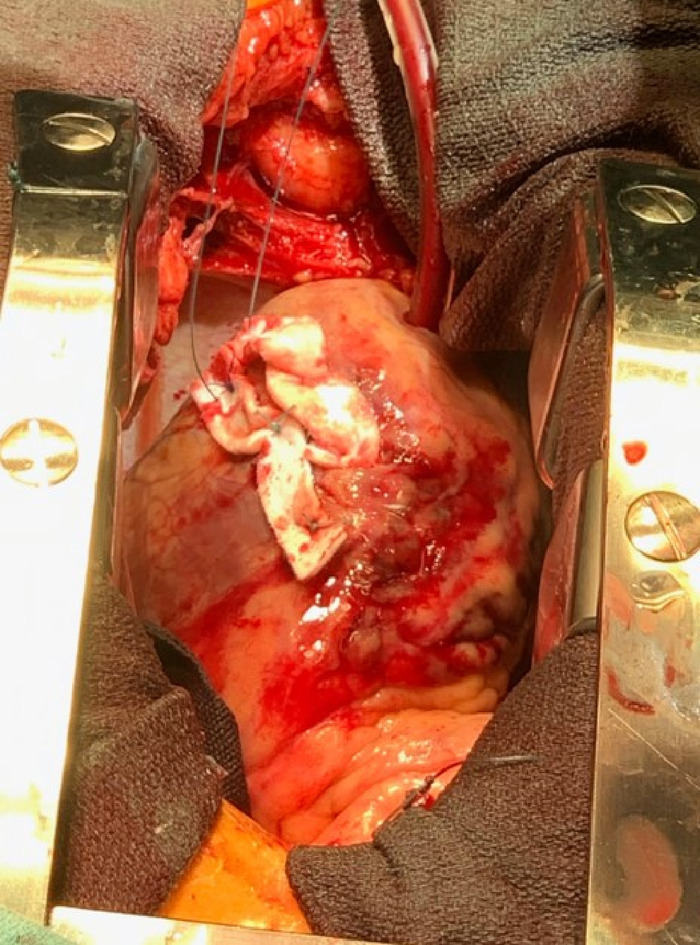
The left ventricular free-wall rupture was repaired using a series of felt strips and pledgeted strips.

The patient was emergently taken to the operating room for a presumptive diagnosis of cardiogenic shock secondary to tamponade from hemopericardium. He was immediately put on cardiopulmonary bypass, and a sternotomy was performed. During the pericardiotomy, a large amount of clot and blood was evacuated. Further visual examination revealed a deep laceration and an adjacent smaller laceration along the anterolateral wall with no abnormalities noted on the remaining myocardium (Figure 4), consistent with early LVFWR. The course of the left anterior descending artery was characterized by an edematous petechiae stained pattern (Figure 5). These findings were consistent with an acute infarct with free rupture of the anterolateral wall. The deep laceration was repaired with felt strips, and a series of pledgeted strips were placed around the adjacent minor laceration (Figure 6). A saphenous vein was grafted to the left anterior descending artery.

Postoperatively, the patient's condition improved, and he was successfully weaned off mechanical ventilation, pressors, and inotropes. He was discharged from the hospital on postoperative day 20. He remained clinically well and asymptomatic at 2-year follow-up.

## DISCUSSION

Our patient presented with delayed STEMI; was hemodynamically unstable; and was in distress, hypotensive, cold, and clammy. These symptoms prompted an urgent transthoracic echocardiogram that revealed a complex pericardial effusion. That finding prompted a CT scan that revealed hemopericardium. This constellation of findings prompted the emergent transfer to the operating room where the LVFWR was identified. This case illustrates the importance of identifying the etiologic driver of shock (cardiogenic vs obstructive) in a patient with STEMI, as this result determines the next steps in time-sensitive management decisions. Cardiogenic shock from obstructive coronary artery disease can be treated in the catheterization laboratory. However, obstructive shock as a result of complex pericardial effusion benefits from surgery.

Following STEMI, an estimated 0.27% to 0.91% of patients develop mechanical complications.^8^ Papillary muscle rupture, ventricular free-wall rupture, and ventricular septal rupture are estimated to occur in 0.05% to 0.26% of patients, 0.01% to 0.52% of patients, and 0.17% to 0.21% of patients, respectively.^5,8^ LVFWR can be categorized into 3 types. Type 1 rupture is an abrupt tear that typically occurs within the first 24 hours of myocardial infarction. Type 2 rupture is a slower tear that occurs with a localized myocardial erosion. Type 3 rupture is a thin-walled aneurysm perforation that usually occurs more than 7 days after myocardial infarction.^9,10^ LVFWR usually occurs within 7 days after myocardial infarction, with the majority of these cases occurring within 24 hours of symptom onset of the myocardial infarction.^5^ In a 2018 series, the mean time to diagnosis of LVFWR was 2.6 days.^11^

Patients classically present with chronic/protracted anginal pain that they have experienced for hours to days. Rupture of the left ventricular free wall occurs in 0.5% of patients following acute myocardial infarction and is associated with 20% mortality from pericardial tamponade.^5^ LVFWR typically develops at the border of normal myocardium and infarcted myocardium. A large, abrupt tear leads to sudden cardiac tamponade, cardiogenic shock, and pulseless electrical activity/cardiac arrest. A smaller, more gradual tear may be contained by thrombus formation or pericardium but causes hemodynamic instability and pericardial effusion as in our patient.^12^ Interestingly, our patient had left ventricular hypertrophy, and his myocardial infarction was so severe that it penetrated the hypertrophied ventricle. Surgical intervention is the definitive therapy to close the tear and prevent recurrent rupture or formation of pseudoaneurysm.^12^ Coronary artery bypass at the time of surgery has been associated with increased anginal relief and improved survival.^13,14^

LVFWR should be suspected in a patient with STEMI when a bedside echocardiogram demonstrates a pericardial effusion, tamponade physiology, epicardial clots, or exudative material in the pericardial space.^15^ However, a tear in the ascending aorta with retrograde extension into the coronary arteries or hemorrhagic pericarditis may also present with a pericardial effusion containing exudative material. After hemodynamic stability was achieved, the patient underwent a CT scan that could not identify the cause of the pericardial effusion but did reveal hemopericardium. At that point, the case was clearly a surgical case, and the patient was rushed to the operating room for mediastinal exploration and repair.

Different opinions have been published about performing coronary angiograms or avoiding them so that treatment and surgery are not delayed.^16,17^ Certainly, having knowledge of coronary status is useful in deciding where to place a bypass graft. In our case, that strategy was abandoned because of the patient's shock and ongoing clinical instability.

Current literature supports cardiogenic shock and LVFWR as major causes of death following STEMI.^3-8,10-17^ The dilemma that providers face when dealing with a hypotensive STEMI patient is trying to identify if the patient has cardiogenic shock from pump failure vs obstructive shock from hemorrhagic pericardium. Cardiogenic shock should be suspected when the echocardiogram reveals reduced left ventricular function and low ejection fraction. LVFWR should be suspected when the echocardiogram demonstrates a complex pericardial effusion with tamponade physiology. In our case, the finding of a complex pericardial effusion plus preserved ejection fraction triggered a series of investigational studies that suggested pathology that could not be solely fixed in a catheterization laboratory but would require surgical investigation and possible intervention. Therefore, the patient was taken to the operating room where the LVFWR was discovered and appropriately treated.

## CONCLUSION

In a patient who presents with signs and symptoms of an acute myocardial infarction and concomitant hemodynamic instability, the possibility of a mechanical complication should remain high on the differential. Echocardiography can quickly identify mechanical complications of myocardial infarctions. If hemodynamically significant pericardial effusion is identified, further investigations should be performed to delineate the cause of the pericardial effusion.

## Notes

*This article meets the Accreditation Council for Graduate Medical Education and the American Board of Medical Specialties Maintenance of Certification competencies for Patient Care and Medical Knowledge*.
